# Antifungal Effect of Vitamin D_3_ against *Cryptococcus neoformans* Coincides with Reduced Biofilm Formation, Compromised Cell Wall Integrity, and Increased Generation of Reactive Oxygen Species

**DOI:** 10.3390/jof9070772

**Published:** 2023-07-21

**Authors:** Jian Huang, Junwen Lei, Anni Ge, Wei Xiao, Caiyan Xin, Zhangyong Song, Jinping Zhang

**Affiliations:** Public Center of Experimental Technology, School of Basic Medical Sciences, Southwest Medical University, Luzhou 646000, China; jane_h9843@163.com (J.H.);

**Keywords:** *Cryptococcus neoformans*, vitamin D_3_, antifungal agent, cell wall, cell membrane, reactive oxygen species

## Abstract

*Cryptococcus neoformans* is an invasive fungus that causes both acute and chronic infections, especially in immunocompromised patients. Owing to the increase in the prevalence of drug-resistant pathogenic fungi and the limitations of current treatment strategies, drug repositioning has become a feasible strategy to accelerate the development of new drugs. In this study, the minimum inhibitory concentration of vitamin D_3_ (VD_3_) against *C. neoformans* was found to be 0.4 mg/mL by broth microdilution assay. The antifungal activities of VD_3_ were further verified by solid dilution assays and “time-kill” curves. The results showed that VD_3_ reduced fungal cell adhesion and hydrophobicity and inhibited biofilm formation at various developmental stages, as confirmed by crystal violet staining and the 2,3-bis(2-methoxy-4-nitro-5-sulfophenyl)-2H-tetrazolium-5-carboxanilide assay. Fluorescence staining of cellular components and a stress susceptibility assay indicated that VD_3_ compromised cell integrity. Reverse transcription quantitative PCR demonstrated that VD_3_ treatment upregulated the expression of fungal genes related to cell wall synthesis (i.e., *CDA3*, *CHS3*, *FKS1*, and *AGS1*). Moreover, VD_3_ enhanced cell membrane permeability and caused the accumulation of intracellular reactive oxygen species. Finally, VD_3_ significantly reduced the tissue fungal burden and prolonged the survival of *Galleria mellonella* larvae infected with *C. neoformans*. These results showed that VD_3_ could exert significant antifungal activities both in vitro and in vivo, demonstrating its potential application in the treatment of cryptococcal infections.

## 1. Introduction

In addition to the serious threats posed by various viral outbreaks and drug-resistant bacterial infections, fungal infections have also received widespread attention in recent years. Fungal diseases affect more than 1 billion people worldwide annually, resulting in over 1.5 million deaths [1]. *Cryptococcus neoformans* is a particularly harmful species of invasive fungi, the latest report estimated that there were 152,000 cases (111,000–185,000) of cryptococcal meningitis (CM), resulting in 112,000 cryptococcal-related deaths (79,000–134,000) globally each year [2]. It is a ubiquitous opportunistic fungal pathogen, and its infection results from the inhalation of airborne spores. While *C*. *neoformans* is rapidly cleared by the immune system in healthy individuals, it can cause lung infections in immunocompromised patients, organ transplant recipients, cancer patients receiving chemotherapy, and individuals with advanced human immunodeficiency virus (HIV) infections. In more serious cases, *C*. *neoformans* can spread to the brain, causing CM [3,4]. HIV-associated CM is responsible for approximately 70% of mortality in developing African countries [5] and 150,000–200,000 deaths worldwide each year [6].

Clinically, the standard treatment regimen for *C*. *neoformans* infection consists of a short course of amphotericin B combined with flucytosine, followed by fluconazole (FCZ) consolidation monotherapy [7]. However, up to 60% of patients experience relapse after the standard treatment regimen, and the clinical resistance to FCZ continues to increase [8]. A previous study reported that up to 25% of colonies derived from clinical isolates were resistant to FCZ, and 11 of 13 patients exhibited heteroresistance within 7 days of FCZ monotherapy [9]. Amphotericin B resistance in *C*. *neoformans* is rare; however, intravenous administration and the high host toxicity limit its use [10]. The limited availability of antifungal agents and the emergence of antifungal resistance highlight the urgent need for new strategies against aggressive fungal infections. Repositioning and re-development of existing drugs, rather than the time-consuming and expensive process of developing new drugs, can offer a quick alternative strategy to address this problem [11,12].

Vitamin D_3_ (VD_3_) regulates calcium and phosphorus homeostasis in bone metabolism and has been suggested to exhibit some antiviral activities. For example, VD_3_ has been reported to attenuate rotavirus infection by regulating autophagy maturation and porcine antimicrobial peptide gene expression [13]. Moreover, VD_3_, which is highly fat-soluble, has been noted to affect the cell membrane integrity of *Candida albicans*, thereby presenting antifungal activities [14]. A previous study by our group confirmed that VD_3_ exhibits significant anti-*C. albicans* activity both in vivo and in vitro [15]. The present study is the first to investigate the antifungal effect and mode of action of VD_3_ against *C. neoformans*. The results obtained confirmed that the antifungal effect of VD_3_ against *C. neoformans* coincides with reduced biofilm formation, compromised cell wall integrity, and increased generation of reactive oxygen species (ROS). Thus, VD_3_ could be a potential antifungal drug for the prevention and treatment of *C. neoformans* infections.

## 2. Materials and Methods

### 2.1. Chemicals, Reagents, and Culture Conditions

VD_3_ (67-97-0, Macklin, Shanghai, China) and 2,3-bis(2-methoxy-4-nitro-5-sulfophenyl)-2H-tetrazolium-5-carboxanilide (XTT) (111072-31-2, Macklin, Shanghai, China) were used in this study. Roswell Park Memorial Institute (RPMI) 1640 medium was obtained from HyClone Laboratories, Inc. (South Logan, UT, USA). Calcium fluoride white (CFW), sodium dodecyl sulfate (SDS), and Congo red were purchased from Sigma-Aldrich Corporation (St. Louis, MO, USA). Before each experiment, a stock solution of VD_3_ dissolved in dimethyl sulfoxide (DMSO) at 50 mg/mL was prepared and diluted to the target concentration with RPMI 1640 medium or YPD medium (1% yeast extract, 2% peptone, and 2% dextrose) containing 0.05% Tween 80. *C*. *neoformans* strain H99 was stored in 30% glycerol at −80 °C and cultured overnight in YPD medium at 37 °C and 200 rpm prior to use.

### 2.2. Broth Microdilution Assay

The minimum inhibitory concentration (MIC) of VD_3_ against *C. neoformans* was determined using the Clinical and Laboratory Standards Institute Standard M27-A3 Reference Method for Broth Dilution Antifungal Susceptibility Testing of Yeasts [16] with minor modifications. Activated *C*. *neoformans* cells were resuspended in RPMI 1640 medium to a final concentration of 5 × 10^3^ cells/mL. Various concentrations of VD_3_ (0.0, 0.05, 0.1, 0.2, 0.3, 0.4, 0.5, 0.6, 0.7, and 0.8 mg/mL) were prepared as described in our previous report [15]. A positive drug-free control and a negative yeast-free control were established. The test strains and controls were incubated at 37 °C for 72 h. The optical density at 600 nm (OD_600_) was measured using a Varioskan™ LUX multimode microplate reader (Thermo Fisher Scientific, Waltham, MA, USA) to determine the minimum concentration needed to inhibit the growth of 90% (MIC_90_) of isolates.

### 2.3. Spot Dilution and Stress Susceptibility Assay

A spot dilution assay was performed to determine the antifungal effect of VD_3_ as described previously [17,18]. In brief, 3-microliter aliquots of *C*. *neoformans* strain H99 at various concentrations (10^2^, 10^3^, and 10^4^ cells/mL) were spotted onto YPD plates containing different concentrations of VD_3_ (0.1, 0.2, 0.3, and 0.4 mg/mL) and incubated at 37 °C for 72 h. In the control group, an equal volume of DMSO was added. After incubation, the plates were photographed using a digital camera (Canon Inc., Tokyo, Japan).

For the stress susceptibility assay, an overnight culture of *C. neoformans* strain H99 was washed and diluted to 1 × 10^7^ cells/mL in phosphate-buffered saline (PBS). The cell wall stress susceptibility was tested using 100 μg/mL CFW, 0.01% SDS, and 0.2% Congo red, as described previously [19] with slight modifications. Sensitivity to oxidative stress was evaluated with 1 mM H_2_O_2_ (Sigma-Aldrich Corporation). In brief, the cell suspensions were serially diluted in YPD medium containing different concentrations of VD_3_ (0, 0.4, and 0.8 mg/mL) with or without the stressors, incubated at 37 °C for 3 days, and photographed.

### 2.4. Time-Kill Assay

The time-kill assay was performed as described previously [15]. An overnight culture of *C*. *neoformans* strain H99 was washed, diluted to 5 × 10^5^ cells/mL in YPD medium containing 0.4 mg/mL VD_3_ or DMSO, and incubated at 37 °C and 200 rpm. A portion of the cell suspension was collected at 2, 4, 8, 12, 16, and 24 h, respectively, diluted with PBS, and spread onto YPD plates. After incubation of the plates at 37 °C for 72 h, the number of colony-forming units (CFUs) was quantified. The experiment was repeated three times on separate days, and the data were averaged for analysis.

### 2.5. Biofilm Inhibition Assay

The effects of VD_3_ on biofilm formation by *C*. *neoformans* strain H99 were assessed by crystal violet (CV) staining and XTT assay [20,21]. In brief, the *C*. *neoformans* cells were washed and diluted to 10^6^ cells/mL in RPMI 1640 medium and incubated in 96-well plates at 37 °C for 90 min (initial phase), 12 h (developmental phase), and 48 h (maturation phase). Furthermore, the medium and non-adherent cells were discarded. Subsequently, RPMI 1640 medium containing 0.4 and 0.8 mg/mL VD_3_ or DMSO was added to the wells and incubated at 37 °C for 6 h. After incubation, the medium was discarded, the cells were rinsed with PBS, and fresh RPMI 1640 medium (200 μL) was added to each well and incubated for 48 h. Moreover, each well was washed twice with PBS, and the biofilm mass and metabolic activity of the cells were measured at OD_595_ and OD_490_, respectively. The biofilm activity was calculated as (OD3 − OB)/(OC − OB) × 100%, where OD3, OC, and OB are the OD values of VD_3_, DMSO, and blank groups, respectively.

The three-dimensional structure of the biofilm produced by *C*. *neoformans* after VD_3_ treatment was visualized using a confocal laser scanning microscope (TCS SP8; Leica Microsystems GmbH, Wetzlar, Germany) [21]. In brief, the yeast cells were washed and diluted to 10^6^ cells/mL in RPMI 1640 medium containing 0.4 mg/mL VD_3_ or DMSO in a 6-well plate and incubated at 37 °C for 48 h. After incubation, the supernatant was discarded, and the cells were rinsed with PBS, stained with CFW at 37 °C for 10 min, and imaged.

### 2.6. Adhesion Assay and Cell Surface Hydrophobicity Analysis

To explore the effect of VD_3_ on the adhesion activity of *C*. *neoformans*, an adhesion assay was performed as previously described [22], with slight modifications. In brief, the yeast cells were diluted to 10^6^ cells/mL in RPMI 1640 medium containing 0.4 and 0.8 mg/mL VD_3_ or DMSO in a 96-well plate and incubated at 37 °C for 4 h. Subsequently, the medium along with non-adherent cells were discarded, the wells were rinsed thrice with PBS, and 200 μL of fresh RPMI 1640 medium were added to each well and incubated for 48 h. Finally, each well was washed twice with PBS. Early adhesion activity was confirmed by CV staining and the XTT assay, similarly. The adhesion activity was calculated as (OD3 − OB)/(OC − OB) × 100%, where OD3, OC, and OB are the OD values of VD_3_, DMSO, and blank groups, respectively.

Cell surface hydrophobicity (CSH) analysis was performed as previously described [23] with some modifications. In brief, the yeast cells were washed and diluted to 10^6^ cells/mL in YPD medium containing 0.4 and 0.8 mg/mL VD_3_ or DMSO in a 96-well plate and incubated for 6 h at 37 °C and 200 rpm. Furthermore, the cells were collected, resuspended in 2.45 mL of PBS, and the absorbance of 200 μL of the cell suspension was determined at OD_600_ (D0). Subsequently, 3 mL of chloroform were added to the cell suspension and mixed for 3 min, and the mixture was allowed to stand for 30 min. After that, the absorbance of 200 μL of the supernatant was measured at OD_600_ (D1). The CSH was calculated as (D0 − D1)/D0.

### 2.7. Cell Wall Staining, Microscopy, and Quantification of Cellular Components

The *C. neoformans* cells treated without or with 0.4 and 0.8 mg/mL VD_3_ were incubated at 37 °C and 200 rpm for 6 h. Furthermore, the cells were washed with PBS, resuspended to a concentration of 5 × 10^7^ cells/mL, fixed with 4% paraformaldehyde at room temperature for 15 min, washed again with PBS, and stained as described previously [24]. The chitooligomers of the cell wall were stained with 100 µg/mL fluorescein isothiocyanate-labeled wheat germ agglutinin (Sigma-Aldrich Corporation, Bengaluru, India) at 37 °C for 35 min in the dark. Chitin was stained by incubating the cells with 5 μg/mL CFW at 37 °C for 10 min in the dark, and chitosan was stained by incubating the cells with 300 μg/mL Eosin Y (Shanghai Macklin Biochemical Co., Ltd., Shanghai, China) at 37 °C for 10 min. After staining, the cells were washed twice with PBS and observed at 40× under a fluorescence microscope (BX63; Olympus Corporation, Tokyo, Japan).

Staining of β-1,3-glucan was performed as described previously [25]. In brief, The *C. neoformans* cells treated without or with 0.4 and 0.8 mg/mL VD_3_ were incubated at 37 °C and 200 rpm for 6 h. Furthermore, the cells were washed with PBS, resuspended to a concentration of 5 × 10^7^ cells/mL, and then the OD_600_ was measured. To determine the total glucan content, cells were stained with 0.1% aniline blue (Wako Pure Chemical Industries, Ltd., Osaka, Japan) at 80 °C for 15 min in the dark. Subsequently, fluorescence was measured at excitation and emission wavelengths of 400 and 460 nm, respectively, using a plate reader. Furthermore, the yeast cells were stained with 5 μg/mL CFW as described previously [26], and the total chitin content was quantified by measuring fluorescence at excitation and emission wavelengths of 365 and 435 nm, respectively, using a plate reader. The change in fluorescence (∆F) was calculated as [F(test) − F(blank)], where F(test) is the fluorescence of the test sample and F(blank) is the fluorescence of the test group without dye solution.

### 2.8. Fungal Membrane Integrity and Intracellular Content of Reactive Oxygen Species (ROS)

The membrane integrity of *C. neoformans* cells was assessed by staining the cells with propidium iodide (PI) (Solarbio Science and Technology Co., Ltd., Beijing, China) as described previously [22]. The *C. neoformans* cells treated without or with 0.4 and 0.8 mg/mL VD_3_ were incubated at 37 °C and 200 rpm for 6 h. Furthermore, the cells were washed with PBS, resuspended to a concentration of 5 × 10^7^ cells/mL, and then the OD_600_ was measured. Cells were stained with 5 μM PI for 10 min at 37 °C in the dark. Following that, the cells were washed with PBS, and the fluorescence was measured at excitation and emission wavelengths of 535 and 617 nm, respectively, using a plate reader, and the cells were observed at 40× under a fluorescence microscope.

The intracellular ROS content was evaluated by staining the yeast cells with 2′,7′-dichlorodihydrofluorescein diacetate (DCFH-DA) (Sigma-Aldrich Corporation) as described previously [27]. In brief, the cells treated without or with 0.4 and 0.8 mg/mL VD_3_ were incubated at 37 °C and 200 rpm for 6 h. Furthermore, the cells were washed with PBS, resuspended to a concentration of 5 × 10^7^ cells/mL, and then the OD_600_ was measured. Cells were stained with 10 μM DCFH-DA for 30 min at 37 °C in the dark. The fluorescence was measured at excitation and emission wavelengths of 485 and 530 nm, respectively, using a plate reader, and the cells were observed at 40× under a fluorescence microscope.

### 2.9. RNA Isolation and Reverse Transcription Quantitative PCR

Overnight cultures of *C. neoformans* (10^6^ cells/mL) were treated with or without 0.4 mg/mL VD_3_ at 37 °C and 200 rpm for 6 h. Furthermore, the cells were collected by centrifugation at 4400 rpm at 4 °C for 3 min. The total RNA was isolated using yeast processing reagent (TaKaRa, Dalian, China), reverse-transcribed into cDNA using PrimeScript™ RT Reagent Kit with gDNA Eraser (Takara Bio, Inc., Shiga, Japan), and amplified by reverse transcription quantitative PCR (RT-qPCR) with TB Green Premix Ex Taq™ Ⅱ Master Mix (Takara Bio, Inc., Dalian, China). The primers used in this study are listed in Supplemental Table S1. The relative expression levels of genes were calculated using 2^−ΔΔCT^ method against glycerol-3-phosphate dehydrogenase 1 (*GPD1*) as the housekeeping gene [24,28].

### 2.10. Antifungal Efficacy of VD_3_ In Vivo

The larvae of the honeycomb moth (*Galleria mellonella*) were used to construct a fungal infection model for survival analysis and the determination of fungal burden [29]. Prior to the experiment, the *G. mellonella* larvae (average body weight, 300 mg) were kept in the dark at 37 °C, and *C*. *neoformans* strain H99 cells were washed, resuspended in normal saline, and diluted to 2 × 10^7^ cells/mL. Moreover, the larvae were infected with 10 μL of *C. neoformans* cells or normal saline (Control group) using a Hamilton syringe. After infection for 2 h, the larvae were injected with 10 μL of the drug or normal saline containing DMSO (Cn group). The experimental groups were treated with 0.5, 1, 5, 10, and 20 mg/kg VD_3_, respectively. The positive control group was treated with 10 mg/kg of FCZ. To quantify the fungal burden, five larvae per group were homogenized after treatment for 24 h, serially diluted 10 folds, inoculated onto YPD plates, and incubated for 72 h at 37 °C. Subsequently, the fungal CFUs were quantified. For survival analysis, 20 larvae per group were monitored daily for 5 days at 37 °C in the dark. A one-way analysis of variance (ANOVA) was used to assess the differences in fungal burden among the groups. Survival curves were analyzed by the Kaplan-Meier method (log-rank test) using GraphPad Prism 9.0 software (GraphPad Software, Inc., San Diego, CA, USA).

### 2.11. Statistical Analysis

The experiments were repeated three times. All statistical analyses were performed using GraphPad Prism 9.0 software. As the data were normally distributed, the differences between the groups were compared with a *t*-test, log-rank test, or one-way ANOVA. A probability (*p*) value < 0.05 was considered statistically significant.

## 3. Results

### 3.1. Antifungal Effects of VD_3_ In Vitro

**Figure 1:**
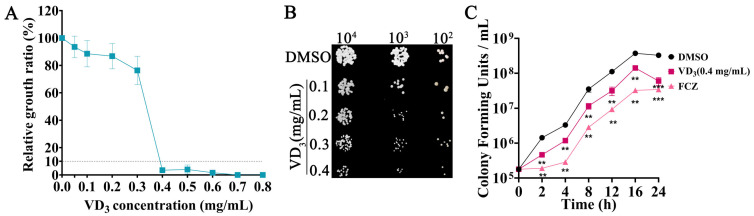
Growth inhibition of *C. neoformans* by VD_3_ in vitro. (**A**) Growth inhibition of *C. neoformans* by VD_3_ is evaluated by the broth microdilution method. (**B**) Growth of *C. neoformans* on solid YPD plates containing different concentrations of VD_3_. (**C**) Time-kill curves of *C. neoformans* (initial inoculum concentration of 10^5^ CFU/mL) treated with 0.4 mg/mL VD_3_. VD_3_, Vitamin D_3_; FCZ, fluconazole. Data were analyzed by one-way ANOVA (**, *p* < 0.01; ***, *p* < 0.001).

A previous study by our group confirmed that VD_3_ had significant antifungal activities against *C. albicans* both in vitro and in vivo [15]. In the present study, the broth microdilution method confirmed that the MIC_90_ of VD_3_ was 0.4 mg/mL against *C. neoformans* (Figure 1A). The results of the spot dilution assay showed that the growth of *C. neoformans* was inhibited by VD_3_ on agar medium (Figure 1B). The “time-kill” curve revealed that VD_3_ hindered the growth of *C. neoformans* in the lag, logarithmic, and stationary phases (Figure 1C). These results indicated that VD_3_ exerted significant antifungal activity against *C. neoformans* in vitro.

### 3.2. VD_3_-Inhibited Biofilm Formation by C. neoformans

**Figure 2:**
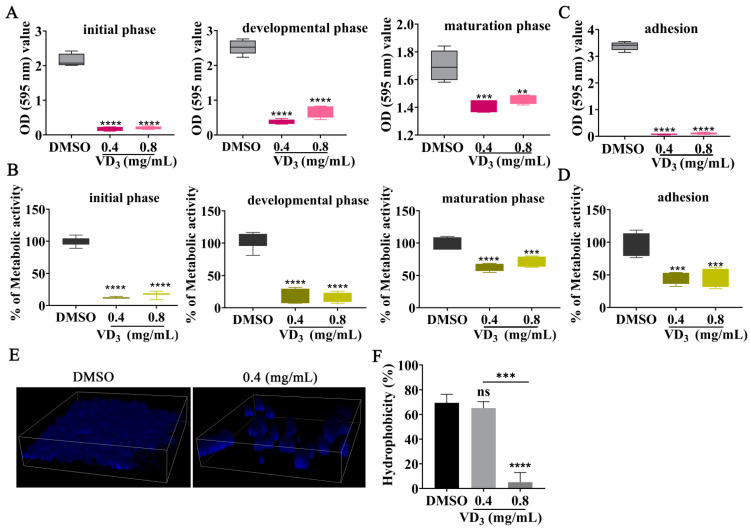
Inhibitory effects of VD_3_ against *C. neoformans* biofilm formation. (**A**) Biomass and (**B**) metabolic activity of *C. neoformans* biofilm at the initial phase (90 min), developmental phase (12 h), and maturation phase (48 h) as determined by CV staining and XTT assay. Adhesion (4 h) activity of *C. neoformans* was evaluated by (**C**) CV staining and (**D**) XTT assay. (**E**) CFW staining of *C. neoformans* cells and the three-dimensional structure of the biofilm. (**F**) Effects of VD_3_ on CSH. Data were analyzed by one-way ANOVA (ns, *p* > 0.05; **, *p* < 0.01; ***, *p* < 0.001; ****, *p* < 0.0001).

A previous investigation found that *C. neoformans* biofilms are resistant to antimicrobial agents and host defense mechanisms, causing significant morbidity and mortality [30]. Therefore, the effects of VD_3_ against biofilm formation by *C. neoformans* were evaluated in the present study. The results showed that VD_3_ significantly inhibited biofilm formation at all fungal growth phases and destroyed mature biofilm (Figure 2A,B). Furthermore, VD_3_ significantly hindered the early adhesion activity of *C*. *neoformans* (Figure 2C,D). Subsequently, the effects of VD_3_ on biofilm structure were assessed using a confocal laser scanning microscope. When compared with the DMSO group, the total amount of *C. neoformans* biofilm was significantly reduced in the VD_3_ groups, and damage to the biofilm was indicated by decreased density and scattered distribution (Figure 2E). Moreover, the CSH analysis results showed that VD_3_ reduced the CSH of *C. neoformans* (Figure 2F).

### 3.3. VD_3_ Impact on Cell Wall Integrity of C. neoformans

**Figure 3:**
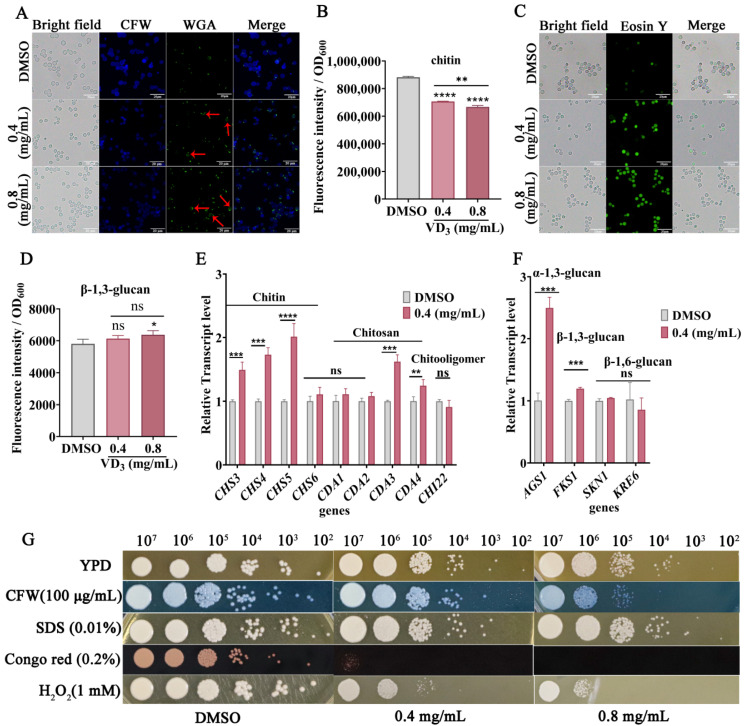
Effects of VD_3_ on cell wall composition and structure of *C. neoformans*. The yeast cells were cultured in YPD medium containing 0.4 and 0.8 mg/mL VD_3_ or DMSO for 6 h. (**A**) Total chitin was stained with CFW, and exposed chitooligomers were stained with fluorescein isothiocyanate-labeled wheat germ agglutinin. *Cryptococcus* cells with altered localization of exposed chitooligomers were indicated by red arrows. (**B**) Total chitin levels of cells stained with CFW were determined using a microplate reader. (**C**) Cell wall chitosan was labeled with Eosin Y and observed under a fluorescence microscope at 40×. Bar, 20 μm. (**D**) The fluorescence intensity of aniline blue was determined to evaluate β-1,3-glucan levels. (**E**) Transcription of genes related to chitin and chitosan syntheses. (**F**) Quantification of glucan biosynthesis by RT-qPCR analysis. The relative expression levels of the genes were calculated using 2^−ΔΔCT^ method against *GPD1* as the housekeeping gene. (**G**) Ten-fold serial dilutions (10^7^, 10^6^, 10^5^, 10^4^, 10^3^, and 10^2^) of *C. neoformans* cells were spotted onto YPD medium containing different concentrations of VD_3_ (0, 0.4, and 0.8 mg/mL) with or without CFW (100 μg/mL), SDS (0.01%), Congo red (0.2%), and H_2_O_2_ (1 mM). The plates were incubated at 37 °C for 72 h. Data were analyzed by one-way ANOVA or *t*-test (ns, *p* > 0.05; *, *p* < 0.05; **, *p* < 0.01; ***, *p* < 0.001; ****, *p* < 0.0001).

The cell wall of *Cryptococcus* is a double-layer structure surrounding the plasma membrane, which is an optimal target for antifungal drugs [31]. Therefore, we are investigating whether VD_3_ impacts cell wall composition and cell wall integrity. When compared with the DMSO group, both CFW fluorescence (Figure 3A) and quantitative results (Figure 3B) demonstrated a decrease in the total chitin content, and chitooligomers staining (by WGA, green fluorescence) was enhanced in the *C. neoformans* cell wall after VD_3_ treatment (Figure 3A), along with localized changes from the cell tip and buds to the entire cell wall (red arrows). In addition, as shown in Figure 3C, VD_3_ groups showed increased staining intensity of chitosan (by Eosin Y, green fluorescence). Meanwhile, β-1,3-glucan levels increased after VD_3_ treatment (Figure 3D). RT-qPCR analysis revealed that the expression levels of *CHS3*, *CHS4*, and *CHS5* (encoding chitin synthase, the key enzyme involved in chitin synthesis), along with *CDA3* and *CDA4* (encoding chitin deacetylase, the key enzyme involved in the synthesis of chitosan from chitin) were significantly upregulated after VD_3_ treatment (Figure 3E); however, there were no significant differences in the expression of *CHI22* (associated with chitooligomer synthesis).

The RT-qPCR results showed that the expression levels of *FKS1* (encoding β-1,3-glucan synthase) and *AGS1* (encoding α-1,3-glucan synthase) were significantly upregulated after VD_3_ treatment, whereas no significant changes were noted in the expression levels of *SKN1* and *KRE6* (involved in the synthesis of β-1,6-glucan) (Figure 3F). Furthermore, VD_3_ treatment altered cell wall structure as detected by staining with CFW, Eosin Y, and WGA, and this coincided with increased susceptibility to cell wall perturbing agents, particularly CFW and Congo red, although there was no increased sensitivity to SDS in combination with VD_3_ (Figure 3G). In short, these results demonstrated that VD_3_ compromised cell wall integrity.

### 3.4. VD_3_-Altered Cell Membrane Permeability of C. neoformans

**Figure 4:**
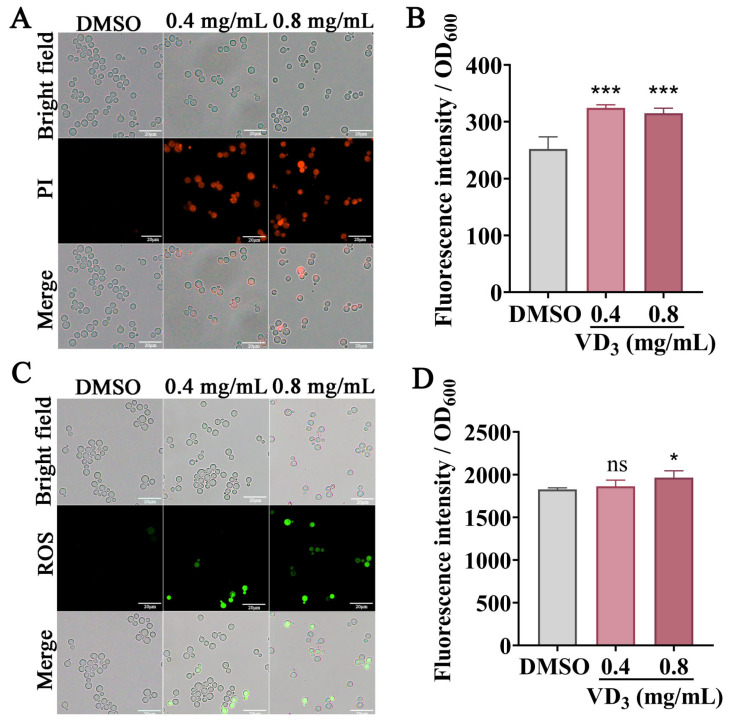
Effects of VD_3_ on *C. neoformans* cell membrane permeability and ROS accumulation. *C. neoformans* cells were treated with or without VD_3_ at 37 °C for 6 h. PI combined with nucleic acids emitted red fluorescence. (**A**) Images captured using a fluorescence microscope. (**B**) Fluorescence was measured using a microplate reader. (**C**,**D**) Green fluorescence of DCFH-DA indicates ROS accumulation. Bar, 20 μm. Data were analyzed by one-way ANOVA (ns, *p* > 0.05; *, *p* < 0.05; ***, *p* < 0.001).

To further explore the structural damage caused by VD_3_, *C. neoformans* cell membrane permeability was evaluated by PI staining. In principle, PI can permeate damaged fungal cell membranes, bind to nucleic acids, and emit red fluorescence [28]. As shown in Figure 4A,B, the untreated fungal cells did not emit red fluorescence, whereas the VD_3_-treated fungal cells emitted strong red fluorescence, with the fluorescence intensity increasing. These results suggested that VD_3_ compromised fungal cell membrane integrity.

### 3.5. VD_3_-Induced Intracellular ROS Accumulation in C. neoformans

A DCFH-DA probe was used to measure the ROS levels in *C. neoformans* cells. As shown in Figure 4C, when compared with the DMSO group, the green fluorescence intensity increased after VD_3_ treatment, especially after 0.8 mg/mL VD_3_ treatment (Figure 4D). Furthermore, hydrogen peroxide can induce cellular oxidative damage; we tested the cellular sensitivity to oxidative stress after VD_3_ treatment. As shown in Figure 3G, VD_3_ significantly restricted cell growth on the agar medium after adding 1 mM H_2_O_2_. These results suggested that VD_3_ induced the accumulation of intracellular ROS and caused oxidation and an antioxidant imbalance in *C. neoformans* cells.

### 3.6. Antifungal Activity of VD_3_ In Vivo

**Figure 5:**
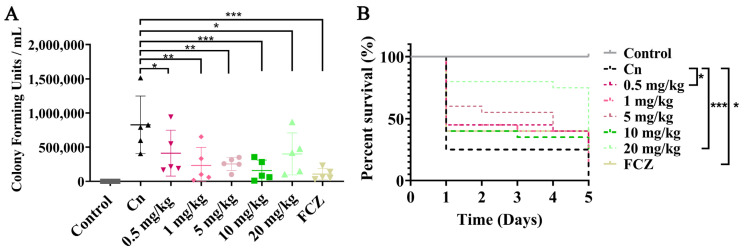
Antifungal effects of VD_3_ in vivo. The *G. mellonella* larvae were infected with 2 × 10^5^ *C. neoformans* cells and treated 2 h later. Control group, larvae injected with normal saline and treated with normal saline containing DMSO; Cn group, larvae injected with *C. neoformans* suspended in normal saline and treated with normal saline containing DMSO; VD_3_ groups, larvae injected with *C. neoformans* and treated with various concentrations of VD_3_ (0.5, 1, 5, 10, and 20 mg/kg, respectively); FCZ group, larvae injected with *C. neoformans* and treated with 10 mg/kg FCZ. The larvae were incubated in the dark at 37 °C. (**A**) The fungal burden in the tissues of *G*. *mellonella* larvae (5 larvae per group) was investigated after 24 h of treatment. (**B**) Larval survival curves (20 larvae per group) after 5 days of treatment. Data on fungal burden were analyzed by one-way ANOVA, and the survival curves were examined using the Kaplan-Meier method (log-rank test) (*, *p* < 0.05; **, *p* < 0.01; ***, *p* < 0.001).

The *G. mellonella* larval infection model is commonly used to study the pathogenesis of *C. neoformans* in vivo [32]. To evaluate the in vivo antifungal effects of VD_3_, the burden of *C. neoformans* in tissues and the survival of infected *G. mellonella* larvae were analyzed. When compared with the *C. neoformans*-infected group, the fungal burden of all VD_3_-treated groups was significantly reduced (Figure 5A). Survival analysis showed that the mortality rate of *G. mellonella* larvae infected with *C*. *neoformans* and without VD_3_ treatment was 75% on day 1, which increased to 100% within 5 days. However, on the fifth day of infection, the survival rates for 0.5, 1, 5, 10, and 20 mg/kg VD_3_ groups and FCZ groups were 30%, 15%, 20%, 20%, 40%, and 30%, respectively. Treatments with 0.5 or 20 mg/kg VD_3_ or 10 mg/kg FCZ significantly prolonged the survival of the larvae (Figure 5B). These results demonstrated that VD_3_ exhibited significant antifungal effects in vivo.

## 4. Discussion

**Figure 6:**
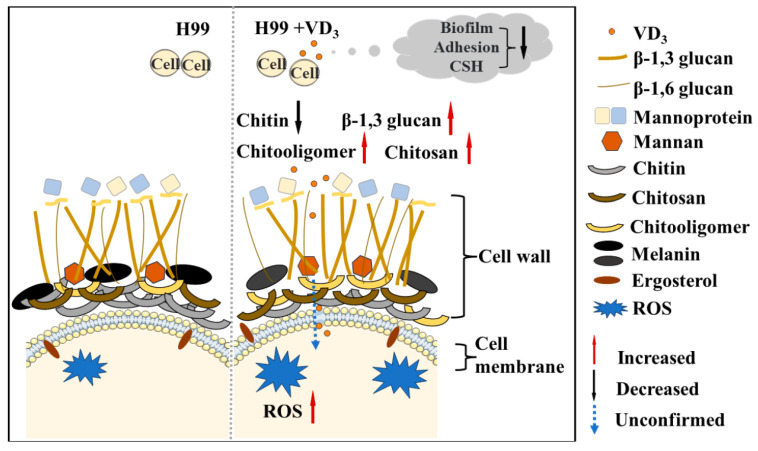
Model: Effects of VD_3_ on *C. neoformans* biofilm, cell integrity, and ROS accumulation. VD_3_ inhibited biofilm formation, hindered early adhesion activity, and reduced the CSH of *C. neoformans*. Furthermore, VD_3_ enhanced cell membrane permeability, impacted cell wall integrity, and caused the accumulation of intracellular ROS. We observed a decrease in cell wall chitin (black solid arrow) and an increase in β-1,3 glucan, chitooligomers, and chitosan staining intensity by VD_3_ (red solid arrow); however, whether VD_3_ can destroy the cell surface and play an antifungal role in the cell remains to be confirmed (blue dotted arrow). CSH, cell surface hydrophobicity; ROS, reactive oxygen species.

The significant increase in *C. neoformans*-related infections and deaths suggests that the development of novel antifungal drugs has not kept pace with the increase in drug resistance among fungi [33]. Consequently, drug repurposing has become a novel research direction [34]. The present study is the first to investigate the antifungal effects and mechanism of VD_3_ against *C. neoformans* infection. The results demonstrated that VD_3_ exhibited significant anti-biofilm activities, compromised the cell wall, enhanced cell membrane permeability, and induced intracellular ROS accumulation in *C. neoformans*. A simplified illustration model with the phenotypic characteristics observed in this study by VD_3_ in *C. neoformans* is summarized in Figure 6.

In this study, VD_3_ increased intracellular ROS levels and significantly increased cell H_2_O_2_ sensibility (Figure 3G). Optimal levels of intracellular ROS ensure appropriate physiological and biochemical activities in the cells, and intracellular ROS signal transduction might possibly be related to the biosynthesis of enzymes involved in maintaining the integrity of the cell wall and cell membrane [35]. However, excessive ROS (i.e., H_2_O_2_, oxides, and hydroxide) accumulation can be toxic to the cell membrane, DNA, and other structures, resulting in abnormal energy metabolism and apoptosis and making cells more susceptible to the environment [36]. The result was consistent with the universal action mechanism of amphotericin B against *C. neoformans* [37]. SDS can disrupt cell membranes and activate cell wall integrity signaling [38]. Interestingly, although there was no effect of VD_3_ on cell sensitivity under 0.01% SDS, we observed that VD_3_ made cells more resistant to high concentrations of SDS on agar medium (Figure S1). This may be caused by VD_3_ interfering with SDS. Further investigations are needed to explore this finding.

During the infection process, invasive pathogenic fungi often produce biofilms to facilitate the production of multiple aggregates of surface-attached cells that promote survival in harsh environments and increase resistance to external stressors [39]. Clinically, *C. neoformans* has also been reported to form biofilms on medical devices [40] and exhibit increased virulence in vivo [41], and its biofilm formation ability is closely related to chronic infection and pathogenesis [42]. Moreover, biofilm formation has been linked to increased fungal resistance to host immunity and antifungal therapies [43]. The formation of biofilm by *C. neoformans* is a complex biological process with coordinated stages, including surface adhesion, microcolony formation, exopolymeric matrix production, and maturation phases [30]. In the present study, VD_3_ exhibited significant antibiofilm activity in *C. neoformans* (Figure 2), similar to that reported in *C. albicans* [15]. In addition, comparable antibiofilm activities have also been exhibited by amphotericin B in fungi [44]. However, the effective concentrations of amphotericin B are considerably toxic, and biofilm formation can significantly reduce the efficacy of antifungal drugs against cryptococcal infections [45]. To resolve these issues, for the treatment of chronic cryptococcal infections, new drugs are often explored in combination with conventional clinical agents to increase their drug susceptibility and reduce their toxicity [46].

The fungal cell wall is a dynamic structure and is considered an ideal target for antifungal drugs [47]. The *C. neoformans* cell wall primarily consists of glucose polymers composed of acetylglucosamine, chitosan, and α-/β-glucans, in addition to mannoproteins [48]. Chitosan is the deacetylation product of chitin, synthesized by enzymes encoded by *CDA*, while chitooligomers are the products of chitin consumption by *CHI22*-encoded endochitinase [25]. No significant changes in the *CHI22* transcription levels were noted, whereas an increase in exposed chitooligomers was observed by fluorescent staining (Figure 3). VD_3_ may increase exposure to chitooligomers by altering the cell wall composition and structure. Abnormally increased exposure to chitooligomers can lead to harmful immune responses [49]. Furthermore, in the present study, upregulation of *CDA3* and *CDA4* involved in the chitosan synthesis pathway led to increased chitosan production, whereas upregulation of *CHS3*, *CHS4*, and *CHS5* involved in the chitin synthesis pathway did not result in increased chitin levels, which might be attributed to insufficient synthesis and excessive consumption of chitin for a long period of time. This imbalance in the regulation of chitin synthesis/degradation can affect cell replication [50]. It must be noted that chitin, glucan, and chitosan are the main antigens on the cell surface. Moreover, fungal pathogens evade host immune recognition by masking β-1,3-glucan on the cell wall surface [51,52]. Thus, further investigations are needed to confirm whether these changes in the fungal cell wall caused by VD_3_ can affect host-pathogen interactions [49]. Moreover, as *C. neoformans* is naturally resistant to echinocandin, which acts on β-1,3-glucan of the cell wall, it is necessary to determine whether the changes in the β-1,3-glucan content of *C. neoformans* after VD_3_ treatment affect the resistance of the fungal cells to echinocandin.

Fungal CSH is an important cellular biophysical parameter that affects cell-cell and cell-surface interactions [53]. High fungal CSH may promote virulence through more complex mechanisms [54]. However, the mechanism of CSH and its direct correlation with the virulence of *C. neoformans* have not been established. In the present study, VD_3_ reduced the CSH of *C. neoformans*, similar to the effect of VD_3_ on *C. albicans* [15]. Melanin is an important component associated with *C. neoformans* virulence [55], and VD_3_ treatment partially decreased melanin production by *C*. *neoformans* on melanin-induced medium when compared with the DMSO group (Figure S2A). In addition, the expression of *LAC2*, which is related to melanin biosynthesis, was also significantly downregulated after VD_3_ treatment (Figure S2B). These results confirmed that VD_3_ partially reduced the virulence of *C. neoformans* through a complex mechanism.

In addition, according to human high VD_3_ supplementation regimens (4000–8000 IU/day) [56], taking large amounts over a long period poses a risk of poisoning. Therefore, in the case of effective therapeutics, reducing the dosage and the course of treatment are recommended. *C*. *neoformans* was significantly inhibited by 0.4 mg/mL VD_3_ in vitro, and in cytotoxicity tests with VD_3_, our investigations confirmed that 0.1–0.6 mg/mL VD_3_ was nontoxic to HepG2 cells in vitro (unpublished data). Moreover, the liver injury was reduced after treatment with 0.6 mg/kg of VD_3_ in mice with a fungal infection [15]. In this study, after treatment of infected *G. mellonella* larvae with 0.5 and 20 mg/kg of VD_3_ for 24 h, the fungal burden was significantly reduced, and the survival of the larvae was significantly prolonged. Further investigations are needed to confirm the antifungal activity and toxicity of 0.5–20 mg/kg VD_3_ in mice. It is presumed that VD_3_ might be rapidly metabolized in vivo into its active form, calcitriol (1,25-dihydroxycholecalciferol), which could induce antifungal effects through regulation of the host immune response [57,58]. In addition, remodeling of the fungal cell wall composition and structure caused by VD_3_ might also induce a host immune response. Therefore, further research is needed to confirm these assumptions and understand the mechanism of the antifungal activities of VD_3_.

## 5. Conclusions

To the best of our knowledge, this study is the first to investigate the antifungal activities of VD_3_ against *C. neoformans* both in vitro and in vivo. The results confirmed that the antifungal effect of VD_3_ against *C*. *neoformans* coincided with reduced biofilm formation, compromised cell wall integrity, increased generation of ROS, and offered new insights into the role of VD_3_ in cell wall remodeling and induction of host immune response. Nonetheless, further in vivo investigations of VD_3_ for the treatment of cryptococcal infections are needed.

## Data Availability

All data generated or analyzed during this study are included in the article and the Supplementary Materials, those are available from the corresponding author upon reasonable request.

## Supplementary Materials

The following supporting information can be downloaded at: https://www.mdpi.com/article/10.3390/jof9070772/s1. Table S1. Nucleotide sequences of primers used for RT-qPCR analysis. Figure S1. VD_3_ reduced the stress of SDS with high concentrations. Figure S2. Effects of VD_3_ on melanin formation.
