# Supplementary material for: Antifungal Effect of Vitamin D3 against Cryptococcus neoformans Coincides with Reduced Biofilm Formation, Compromised Cell Wall Integrity, and Increased Generation of Reactive Oxygen Species
Source: J Fungi (Basel). 2023 Jul 21;9(7):772. doi: 10.3390/jof9070772 (PMC10381739; doi:10.3390/jof9070772)
Supplement: Supplementary file 1 [file jof-09-00772-s001.zip › jof-2500415-supplementary.pdf]

# Supplementary Material

## Antifungal Effect of Vitamin $D_3$ against *Cryptococcus neoformans* Coincides with Reduced Biofilm Formation, Compromised Cell Wall Integrity, and Increased Generation of Reactive Oxygen Species

**Jian Huang, Junwen Lei, Anni Ge, Wei Xiao, Caiyan Xin, Zhangyong Song * and Jinping Zhang ***

Public Center of Experimental Technology, School of Basic Medical Sciences, Southwest Medical University, Luzhou 646000, China; jane_h9843@163.com (J.H.)

* Correspondence: szy83529@163.com (Z.S.); zhangjpwcx@163.com (J.Z.)

**Table S1.** Nucleotide sequences of primers used for RT-qPCR analysis.

| Gene | Primer Sequence (5'-3') |
|---|---|
| *GPD1 F* | AGTATGACTCCACACATGGTCG |
| *GPD1 R* | AGACAAACATCGGAGCATCAGC |
| *CHS3 F* | ACCCAGGTCTGGCATTCC |
| *CHS3 R* | AGGATCAACATTGGAAGC |
| *CHS4 F* | CGGTCTTCAGGCATTGATTT |
| *CHS4 R* | TTCGGAGTGAAGTGATGCTG |
| *CHS5 F* | GCTTGGATGATCTTCTATATCTG |
| *CHS5 R* | TACCTTCATCATGGATGACA |
| *CHS6 F* | TTGACCCTTGGCACATCT |
| *CHS6 R* | GTTGGCATAAGTATCCTT |
| *CDA1 F* | TCGAGCTATTGCTGCTCAGA |
| *CDA1 R* | GCTGGTAGATGTCGTGCTCA |
| *CDA2 F* | GTAACGAGGTCGTCTTTG |
| *CDA2 R* | TGTAGTTGGTGAGCTCGT |
| *CDA3 F* | ATGTGGCCGATGCTTTTAAC |
| *CDA3 R* | GAAGTGAGAAGGCCTGTTGG |
| *CDA4 F* | CGAACTCTCCAAGGTCGAAG |
| *CDA4 R* | GCGTCTCCAGTGTCATCAGA |
| *CHI22 F* | AATGTGAGTGGGTCGGAAAG |
| *CHI22 R* | TAGCCGCCGAGTCTGTAGAT |
| *AGS1 F* | ATCCTTATCCGTTATTCC |
| *AGS1 R* | AGCTGTTCCTCTAGCGAGC |
| *FKS1 F* | TGGACTGGTGTTTGGTTCAA |
| *FKS1 R* | GTACAAAAGACCGTACTTG |
| *SKN1 F* | CTGGACAATGTATGCGGATG |
| *SKN1 R* | TCCGCAGTGGGATAATCTTC |
| *KRE6 F* | GTCTCGGAAGGCGACTCAT |

| | |
|---|---|
| *KRE6 R* | TCAACTCATTCTTTGGGAAGG |
| *LAC1 F* | TACAACTTTCCCCGACCTC |
| *LAC1 R* | GATGGAGAAGGTGAGCGTC |
| *LAC2 F* | TGTATGGCGCAAGGGGTTACT |
| *LAC2 R* | AGAACACGACTCTCCAAAGC |

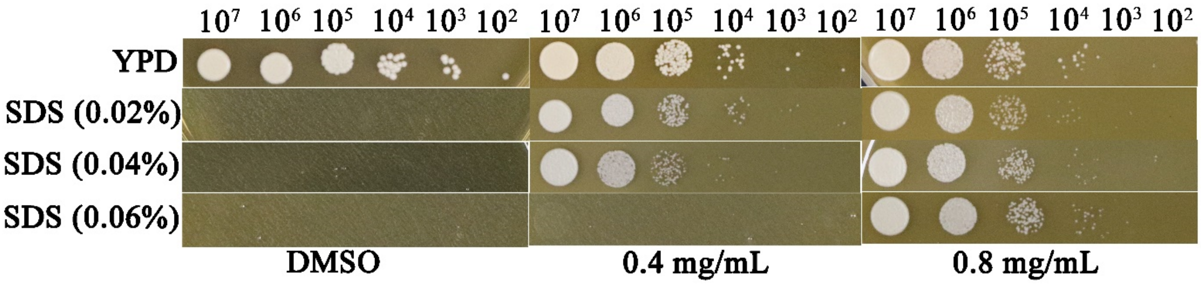


**Figure S1.** VD3 reduced the stress of SDS with high concentrations. An overnight culture of *C. neoformans* strain H99 was washed and diluted to $1 \times 10^7$ cells/mL in phosphate-buffered saline (PBS). Sensitivity to cell surface stress was evaluated with 0.02%, 0.04%, 0.06% SDS (Sigma-Aldrich Corporation). In brief, the cell suspensions were serially diluted in YPD medium containing different concentrations of VD3 (0, 0.4 and 0.8 mg/mL) with or without the stressors, incubated at 37°C for 3 days, and photographed.

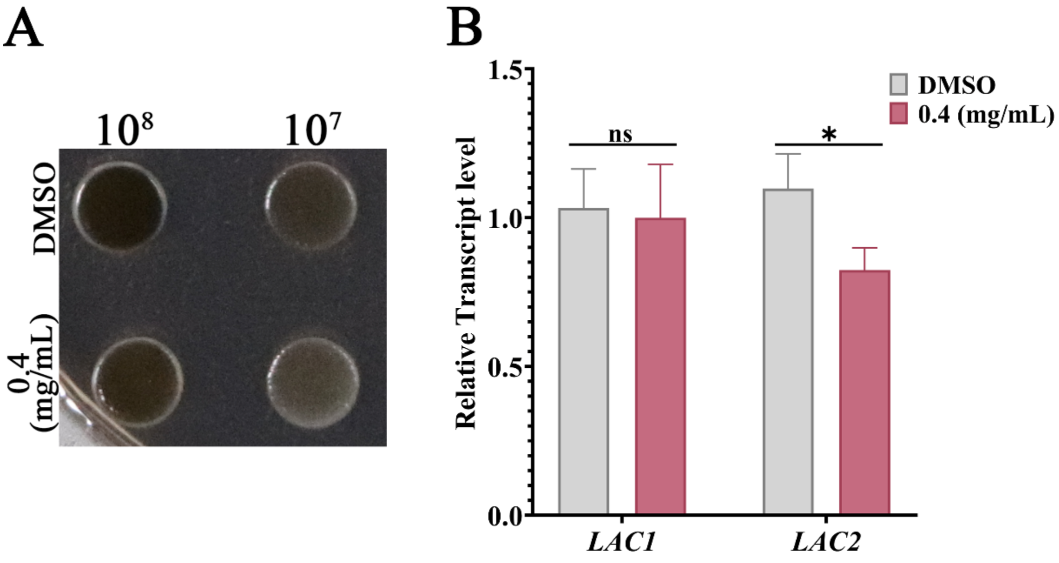


**Figure S2.** Effects of VD3 on melanin formation. *C. neoformans* was cultured in YPD medium containing 0.4 mg/mL DMSO or VD3 for 6 h. (A) VD3 inhibited melanization on minimal medium containing 0.5 mM L-DOPA (3,4-dihydroxy-L-phenylalanine; Sigma-Aldrich Corporation). Images were captured after incubation for 3 days. (B) RT-qPCR quantification of the expression of genes related to melanin biosynthesis. Relative expression levels of genes were calculated using $2^{-\Delta\Delta CT}$ method against *GPD1* as the housekeeping gene. Data were analyzed using t-test (ns, $p > 0.05$; *, $p < 0.05$; **, $p < 0.01$; ***, $p < 0.001$; ****, $p < 0.0001$).
